# Role of circular RNAs and gut microbiome in gastrointestinal cancers and therapeutic targets

**DOI:** 10.1016/j.ncrna.2023.12.002

**Published:** 2023-12-15

**Authors:** Sara Tharwat Abdullah, Snur Rasool Abdullah, Bashdar Mahmud Hussen, Yousif Mohammed Younis, Mohammed Fatih Rasul, Mohammad Taheri

**Affiliations:** a Department of Pharmacology and Toxicology, College of Pharmacy, Hawler Medical University, Erbil, Iraq; b Medical Laboratory Science, College of Health Sciences, Lebanese French University, Kurdistan Region, Erbil, Iraq; c Department of Biomedical Sciences, College of Science, Cihan University-Erbil, Kurdistan Region, 44001, Iraq; d Department of Clinical Analysis, College of Pharmacy, Hawler Medical University, Kurdistan Region, Erbil, Iraq; e Department of Nursing, College of Nursing, Lebanese French University, Kurdistan Region, Erbil, Iraq; f Department of Pharmaceutical Basic Science, Faculty of Pharmacy, Tishk International University, Erbil, Kurdistan Region, Iraq; g Institute of Human Genetics, Jena University Hospital, Jena, Germany; h Urology and Nephrology Research Center, Shahid Beheshti University of Medical Sciences, Tehran, Iran

**Keywords:** Circular RNAs, Gut microbes, Gastrointestinal (GI) cancers, Therapeutic target

## Abstract

Gastrointestinal cancers are a huge worldwide health concern, which includes a wide variety of digestive tract cancers. Circular RNAs (circRNAs), a kind of non-coding RNA (ncRNAs), are a family of single-stranded, covalently closed RNAs that have become recognized as crucial gene expression regulators, having an impact on several cellular functions in cancer biology. The gut microbiome, which consists of several different bacteria, actively contributes to the regulation of host immunity, inflammation, and metabolism. CircRNAs and the gut microbiome interact significantly to greatly affect the growth of GI cancer. Several studies focus on the complex functions of circRNAs and the gut microbiota in GI cancers, including esophageal cancer, colorectal cancer, gastric cancer, hepatocellular cancer, and pancreatic cancer. It also emphasizes how changed circRNA expression profiles and gut microbiota affect pathways connected to malignancy as well as how circRNAs affect hallmarks of gastrointestinal cancers. Furthermore, circRNAs and gut microbiota have been recommended as biological markers for therapeutic targets as well as diagnostic and prognostic purposes. Targeting circRNAs and the gut microbiota for the treatment of gastrointestinal cancers is also being continued to study. Despite significant initiatives, the connection between circRNAs and the gut microbiota and the emergence of gastrointestinal cancers remains poorly understood. In this study, we will go over the most recent studies to emphasize the key roles of circRNAs and gut microbiota in gastrointestinal cancer progression and therapeutic options. In order to create effective therapies and plan for the future gastrointestinal therapy, it is important to comprehend the functions and mechanisms of circRNAs and the gut microbiota.

## 1 Introduction

Gastrointestinal (GI) cancers refer to a variety of malignancies that can appear throughout the digestive system, including the organs that aid in digestion [1]. GI malignancies, such as esophagus cancer [2], gastric cancer [3], hepatocellular cancer [4], colorectal cancer [5], and pancreatic cancer [6], are only a few of the numerous GI malignancies that seriously endanger world health. Research in recent years has revealed that two new variables play a role in the onset and spread of GI malignancies: the circular circRNAs [7] and the gut microbiome [8].

Circular RNAs (circRNAs) are closed and single-stranded RNA molecules and do not have poly (A) tails or 5′–3′ ends [9]. They are more stable and may have a long lifespan within cells because they can withstand the destruction caused by exonucleases [10]. CircRNAs have attracted attention recently due to their variety of roles in gene regulation, like serving as microRNA sponges [11,12], regulating RBPs [13], and even producing functional peptides [14]. They can affect the growth of cancer by controlling important signaling pathways, which play a role in cell differentiation, proliferation, EMT, metastasis, and cell death [[15], [16], [17]].

The gut microbiota has also been found to be a significant contributor to GI malignancies, which refers to the millions of bacteria that survive in the gastrointestinal system of humans [18,19]. The host immune system [20], food metabolism [21], and a variety of bioactive metabolites produced by the gut microbiota all interact with one another to influence the gut microenvironment and general health [22]. Dysbiosis, an imbalance in the gut's microbial population, has been connected to several gastrointestinal illnesses, including esophageal cancer [23], gastric cancer [24], hepatocellular cancer [25], pancreatic cancer [26], and colorectal cancer [27]. The gut microbiota can either directly or indirectly influence tumor development and therapeutic response in GI malignancies [19]. For instance, some gut bacteria can create genotoxic byproducts like nitrosamines or secondary bile acids that can cause DNA damage and encourage the development of cancer [28].

In the context of GI malignancies, the interaction between circRNAs and the gut microbiota has become an exciting topic of investigation, affording unique insights into both disease causes and prospective therapeutic approaches [29]. It may be possible to modify oncogenic pathways by targeting particular circRNAs, and probiotics and dietary changes that aim to alter the gut microbiota may help lower the risk of cancer and improve the effectiveness of current cancer treatments [30,31].

The relationship underlying circRNAs, gut microbiota, and the development of gastrointestinal malignancies is remained unclear, despite significant studies in this direction. In this review, we will discuss the most up-to-date research that focuses on the significance of circRNAs and the gut microbiota in the development and therapeutic interventions of gastrointestinal cancer.

## 2 Role of circRNAs in GI malignancies

The significance of circRNAs in gastrointestinal malignancies is becoming increasingly obvious. Through "sponging" on various miRNAs and altering various signaling pathways, they alter the expression of several classes of genes. Some of these alterations contribute to the development and spread of GI cancer, whereas others do the opposite. The oncogenic circ 0000654 in esophageal squamous-cell carcinomas (ESCCs) promotes ESCC proliferation by sponging miR-149-5p and activating the IL-6/STAT3 signaling cascade [32]. Similarly, He et al. in their in vivo study have shown that circ 0006282, by sponging miR-155, enhances FBXO22 expression and the advancement of GC [33]. Further, Zhang and his team proved that the circTMEM45A expression is increased and acts as an oncogene in hepatocellular cancer (HCC). By mechanically sponging of miR-665 expression, circTMEM45A promotes the upregulation of IGF2 and speeds up the development of HCC [34].

**Fig. 1:**
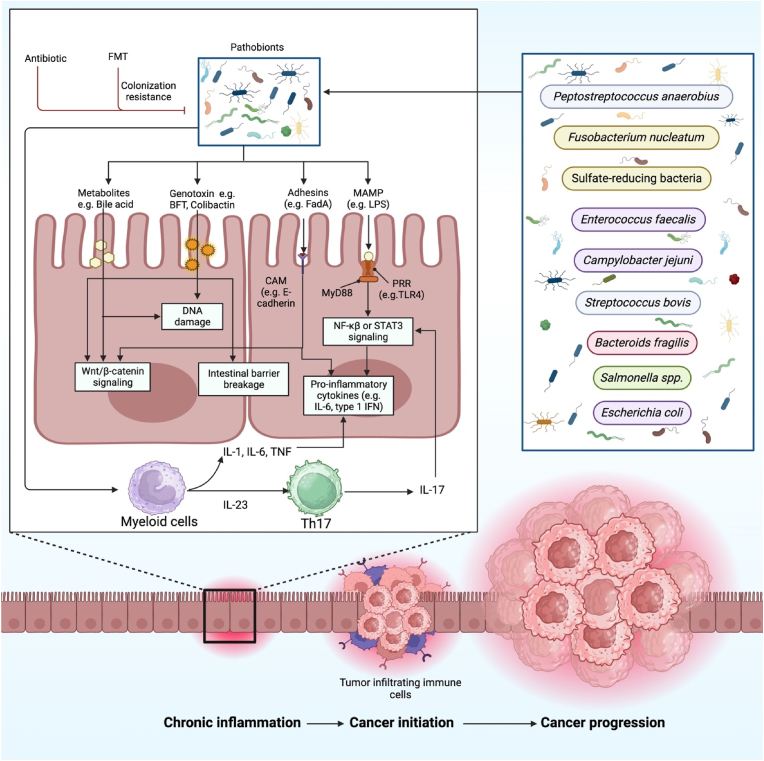
The schematic illustration represents the connection between colorectal cancer's sequential progression and the gut microbiome. Interactions between the host and the microbe lead to activate pro-carcinogenic signaling pathways, which in turn cause molecular changes that speed up CRC. In the colorectal epithelium, certain gut microbes, including *Peptostreptococcus anaerobius, Fusobacterium nucleatum,* sulfate-reducing bacteria, *Enterococcus faecalis*, *Campylobacter jejune*, *Streptococcus bovis*, *Bacteroids fragilis*, *Salmonella* spp., and *Escherichia coli,* cause persistent inflammation. Through changes in several signaling pathways and the production of proinflammatory cytokines, these microbial elements damage DNA and break down the barrier in the gut. Nevertheless, several treatments, such as antibiotics and FMT, inhibit the growth of microbes and colonization.

Although circRNAs are involved in many processes, some may even serve as oncogenes in gastrointestinal malignancies. They play key roles in cancer-related activities including tumor formation and metastasis (Table 1). Through several specific mechanisms oncogenic circRNAs has the ability to control gene expression, such as competing endogenous RNAs (ceRNAs) [35]. CeRNA activity promotes carcinogenesis by elevating carcinogenic mRNAs that miRNAs would typically target and suppress [36]. For instance, in ESCC, Shi et al. revealed that circ_LRP6 sponges miR-198 to induce the invasion, migration, and metastasis of EC [37]. By sponging miR-582-3p, circSHKBP1 was able to upregulate HUR expression, which in turn improved VEGF mRNA stability. Furthermore, circSHKBP1 directly attached to HSP90 and impeded the engagement of STUB1 with HSP90, which in turn inhibited the ubiquitination process of HSP90 and increased the growth of GCs in vitro and in vivo [38]. Similarly, Lu and his colleagues found that circ-RanGAP1 controls the expression of VEGFA by sponging miR-877–3p to promote invasion and metastasis of the GC [39]. Likewise, in CRC cells, Zhang et al. showed that circ-0084615 is an oncogenic circRNA that competes with endogenous RNA to control the expression of *DNMT3A* by miR-599 sponging [40]. Moreover, Xing et al. confirmed that the circular RNA ADAM9 promotes malignant behaviors in pancreatic cancer by sponging miR-217 and elevating PRSS3 expression [41]. In addition, Guo et al. reported that circBFAR enhanced the proliferation of PDAC cells by sponging miR-34b-5p, which increased MET expression and then activated the downstream phosphorylation of Akt (Ser 473) [42]. Furthermore, Liu et al. observed that PC development is aided by the modification of the miR-96-5p/KRAS/MAPK axis, which suggests that the specifically overexpressed has-circ-0006117 may be a potential target for PC therapy [43] (Fig. 1). In CRC, circSPARC was shown to be upregulated in both the plasma and tissues of CRC patients. Mechanistically, circSPARC sponged miR-485-3p to increase JAK2 expression, which in turn helped phosphorylated (p)-STAT3 to be accumulate. Thus, by controlling the JAK/STAT pathway, the circSPARC promotes CRC migration and proliferation [44]. Moreover, Zhang and his colleagues demonstrated that circNRIP1 sponges miR-149-5p to modify AKT1 expression levels and ultimately functions as a tumor promoter in GC. By modulating the AKT1/mTOR axis, circNRIP1 can change metabolism and autophagy and facilitate tumor spread through exosome communication [45].

**Table 1:** Oncogenic roles of various circRNAs in GI tumorigenesis through regulation of target genes and signaling pathways (↑: upregulated, ↓: downregulated).

Cancer types	CircRNAs	Clinical studies	Animal studies	Cell line studies	Target genes/signaling pathways	Clinicopathological characteristics	Description	Ref.
Esophageal cancer	LPAR3	ESCC = 52 case	BALB/c nude mice	ECA109, HET-1A, TE-13, Kyse150, Kyse510, Kyse450	-miR-198, MET -RAS/MAPK and PI3K/Akt axis	Clinical stage and LNM	↑ LPAR3, ↓ miR-198, ↑ MET, ↑ RAS/MAPK and PI3K/Akt signaling: ↑ Metastasis	[37]
	CircAKT3	EC = 82 case	Nude mice	KYSE-150, HEK-293T, TE-10, TE-1	-miR-17-5p, RHOC, STAT3	Tumor size, clinical tumor node metastasis staging, and lymphatic metastasis	↑ CircAKT3, ↓ miR-17-5p, ↑ RHOC*,* STAT3: ↑ Cell proliferation, and metastasis	[51]
	Circ_LRP6	ESCC = 78 pairs	Nude mice	TE-1, EC109	-miR-182, Myc	Larger tumor size, later TNM stage	↑Circ-LRP6, ↓ miR-182, ↑ Myc: ↑ Tumor progression	[52]
	hsa_circ_0067934	EC = 51 pairs	–	TE-13, KYSE-410	–	T stage, tumor differentiation, TNM stage	↑hsa_circ_0067934: ↑ Cell proliferation	[53]
	hsa_circ_0000654	ESCC = 55 case	BALB/c athymic nude mice	TE-1, HEEC, KYSE410, TE-13, KYSE45, ECA-109	-miR-149-5p, IL-6, STAT3	Higher T stage, local LNM	↑hsa_circ_0000654, ↓ miR-149-5p, ↑ IL-6/STAT3: ↑ Cell proliferation, and metastasis	[32]
	Circ_0006168	-EC at phase I + II = 17 case - EC at phase III + IV = 20 case	–	ECA-109, HET-1A, KYSE-510	miR-384, RBBP7	–	↑Circ_0006168, ↓miR-384, ↑RBBP7: ↑Cell proliferation, metastasis, and glycolysis	[54]
	CircFNDC3B	EC tissues = 23 pairs	–	ECA109, KYSE150	FNDC3B	–	↑CircFNDC3B, ↑FNDC3B: ↑Proliferation and metastasis	[55]
	hsa_circ_0000277	ESCC tissues = 92 pairs	BALB/c nude mice	ECA109, Het1A, KYSE-410, EC9706, TE-1, KYSE-150, TE-10	miR-4766-5p, LAMA1	Advanced TNM stage, dismal prognosis	↑hsa_circ_0000277, ↓miR-4766-5p, ↑LAMA1: ↑ ESCC progression	[56]
	CircNTRK2	ESCC tissues = 56 pairs	BALB/c nude mice	Eca-109, Het-1A, EC-9706, TE-1, KYSE-150, KYSE-30	miR-140-3p, NRIP1	Advanced TNM stage, LNM	↑CircNTRK2, ↓ miR-140-3p, ↑ NRIP1: ↑ ESCC development	[57]
Gastric cancer	CircPVT1	–	–	MGC-803, AGS	miR-125 family	Sex, age, tumor site, tumor size, differentiation grade, lymph node status, distant metastasis, TNM stage, T stage, lymphatic invasion, nervous invasion	↑ CircPVT1, ↓ miR-125 family: ↑ Cell proliferation.	[58]
	hsa_circ_0000745	GC = 60 pairs	–	–	–	Lymphatic metastasis, differentiation, TNM stage, sex, age	↓hsa_circ_0000745: ↑Gastric growth	[59]
	Circ_RanGAP1	-GC tissue = 97 pairs. - GC plasma exosome = 30 case	BALB/c (nu/nu) mice	HGC-27, AGS, MGC-803, GES-1, MKN45, KATOIII, BGC-823	VEGFA, miR-877-3p	Advanced TNM stage, LNM, and worse survival	↑Circ-RanGAP1, ↓ miR-877-3p, ↑ VEGFA: ↑ Metastasis	[39]
	CiRS-7	-GC tissue = 102 pairs.	BALB/c nude mice	HGC-27, MGC-803, GES-1	-miR-7 -PTEN/PI3K/AKT signaling	Tumor stages, distant metastasis, lymph node involvement, overall survival	↑CiRS-7, ↓ miR-7, ↓PTEN: ↑ GC progression.	[60]
	CircAKT3	GC tissues = 149 (cohorts 1, 2)	BALB/c nude mice	SGC7901, BGC823	PIK3R1, miR-198	Clinical stage, tumor size, histological grade	↑CircAKT3, ↓miR-198, ↑PIK3R1: ↑ resistance to CDDP	[61]
	CircDLG1	GC tissues = 126 case	BALB/c nude mice, C57BL/6 mice	HGC27, HEK293 T, BGC823, MFC, MKN45, MKN28, GES-1, SGC7901, AGS	miR-141-3p, CXCL12	Age, Lauren's classification, sex, tumor size, peritoneal metastasis, tumor cell differentiation	↑CircDLG1, ↓miR-141-3p, ↑CXCL12: ↑ GC progression and anti-PD-1 resistance	[62]
	CircNRIP1	Tissue samples	BALB/c nude mice	BGC-823, GES-1, SGC-7901, HGC-27, MGC-803, AGS, MKN-45	-miR-149-5p -AKT1/mTOR signaling	Lymphatic invasion, tumor size,	↑CircNRIP1, ↓miR-149-5p, ↑AKT1: ↑ Proliferation, invasion, and migration	[45]
	hsa_circ_0008035	GC = 30 case	–	BGC-823, SGC-7901, GES-1, MGC-803, AGS	miR-375, YBX1	–	↑hsa_circ_0008035, ↓miR-375, ↑YBX1: ↑ Tumorigenesis	[63]
	Circ_0006282	Tissue samples	BALB/c nude mice	BGC-823, GES-1, MKN-45, HGC-27, AGS	miR-155, FBXO22	Tumor size, TNM stage, lymph node metastasis	↑Circ_0006282, ↓miR-155, ↑FBXO22: ↑ GC progression	[33]
	hsa_circ_0001368	GC tissue = 68 pairs	BALB/c (nu/nu) mice	HGC-27, HEK 293T, MGC-803, GES1, AGS, NUGC-3,	miR-6506–5p, FOXO3	–	↓hsa_circ_0001368, ↓miR-6506–5p, ↑FOXO3: ↓ GC progression	[64]
Colorectal cancer	Circ_0084615	CRC = 50 case	BALB/c athymic nude mice	HCT116, FHC, SW480, RKO, DLD1	miR-599, DNMT3A	Age, gender, TNM stage, CEA, CA19-9, differentiation	↑Circ_0084615, ↓ miR-599, ↑DNMT3A: ↑ CRC proliferation and metastasis	[40]
	CircSPARC	CRC = 84 case	BALB/c female nude mice	HCT116, LoVo, SW620, HT-29, SW480, DLD1	-miR-485-3p, p-STAT3 -JAK/STAT signaling	Advanced TNM stage, poor survival, and LNM	↑CircSPARC, ↓miR-485-3p, ↑JAK2: ↑ CRC proliferation and migration	[44]
	CircGLIS2	CRC tissue = 3 pairs	–	NCM460, HCT-15, DLD1, HT-29, HCT-8, HCT116, RKO,	- miR-671 -NF-κB signaling	–	↑CircGLIS2, ↓miR-671, ↑ NF-κB signaling: ↑ CRC pro-metastasis microenvironment	[65]
	CircVAPA	CRC tissue = 60 pairs	–	HEK-293T, RKO, SW480, LoVo, SW620, HT29, HCT116,	miR-101	Gender, age, tumor site, tumor size, lymphovascular invasion, differentiation, TNM stage, LNM	↑CircVAPA, ↓miR-101: ↑ CRC progression	[66]
	hsa_circRNA_102958	CRC = 58 case	BALB/c nude mice	LoVo, FHC, SW480, HCT116, SW620, HCT8, HT29	miR-585/CDC25B	Tumor stage, LNM, differentiation, survival rate	↑hsa_circRNA_102958, ↓miR-585, ↑CDC25B: ↑ Tumorigenesis	[67]
	hsa_circ_0007142	Tissue sample	–	HT-29, HCT-116, LoVo, HCO	miR-103a-2-5p	Lymphatic metastasis and poor differentiation	↑hsa_circ_0007142, ↓miR-103a-2-5p: ↑ CRC proliferation and migration	[68]
	CircSEMA5A	–	Nude mice	LoVo, NCM460, SW620, SW480, Caco-2	miR-195-5p, CCNE1	–	↑CircSEMA5A, ↓miR-195-5p, ↑CCNE1: ↑ CRC development	[69]
	Circ-METTL9	–	Nude mice	–	miR-551b-5p, CDK6	–	↑Circ-METTL9, ↓miR-551b-5p, ↑CDK6: ↑ CRC development	[70]
	CircLDLR	CRC tissue = 5 pairs	BALB/c nude mouse	HEK293T, Caco-2, HCT116, HT29, SW480, RKO, LoVo, SW620, HCT8,	miR-30a-3p/SOAT1	TNM stage, overall survival	↑CircLDLR, ↓miR-30a-3p, ↑SOAT1: ↑ CRC progression	[71]
Hepatocellular carcinoma	CircRHOT1	HCC tissue = 100 pairs	Nude mice	Huh7, Hep3B	NR2F6	Overall survival	↑CircRHOT1, ↑NR2F6: ↑ HCC progression	[72]
	CircTMEM45A	HCC tissue = 68 pairs	BALB/c nude mice	LO2, Hep3B, HLE, Huh7, HCCLM6, BEL7402, HCCLM3, MHCC97H, SMCC7721, MHCC97L	miR-665, IGF2	Poor prognosis, age, gender, HBsAg, cirrhosis, tumor size, tumor number, TNM stage, vascular invasion	↑CircTMEM45A, ↓miR-665, ↑IGF2: ↑ HCC progression	[34]
	hsa_circ_0016788	HCC tissue = 63 pairs	BALB/c nude mice	HepG2, LO2, Hep3B, MHCC97L, HCCLM3, Huh7	miR-486, CDK4	–	↑hsa_circ_0016788, ↓miR-486, ↑CDK4: ↑ HCC tumorigenesis	[73]
	Circ-BIRC6	HCC tissue = 55 pairs	BALB/c nude mice	HepG2, L02, Bel-7402, Rockville, Huh-7, MD, SMMC-7721, USA	miR-3918, Bcl2	Age, serum alpha-fetoprotein level, gender, tumor size, TNM stage, vascular invasion	↑Circ-BIRC6, ↓miR-3918, ↑Bcl2: ↑ HCC progression	[74]
	hsa_circRNA_103809	HCC = 60 case	BALB/c nude mice	LO2, HCCLM3, MHCC97L, Hep3B, SK‐HEP‐1, Huh7	miR-377-3p, FGFR1, ERK, cyclin D1, CDK4, CDK6	Age, AFP, HBsAg, cirrhosis, tumor size, Edmonson grade	↑hsa_circRNA_103809, ↓miR-377-3p, ↑FGFR1, ↑ERK: ↑ HCC development	[75]
	CircMAT2B	–	Nude mice	Huh7, HepG2	miR-338 -3p, PKM2	Tumor size, Edmonson stage, vascular invasion, TNM stage, tumor encapsulation, tumor multiplicity	↑CircMAT2B, ↓miR-338-3p, ↑PKM2: ↑ HCC glycolysis and malignancy	[76]
	hsa_circ_0000673	HCC = 51 case	BALB/c (nu/nu) mice	Huh7, Hep3B	miR-767-3p, SET	Overall survival	↑hsa_circ_0000673, ↓miR-767-3p, ↑SET: ↑ HCC malignancy	[77]
	Circ_0000517	HCC = 45 case	BALB/c nude mice	THLE-2, HCCLM3, Huh7	miR-326, IGF1R	–	↑Circ_0000517, ↓miR-326, ↑IGF1R: ↑ HCC development	[78]
	CircMAST1	HCC = 39 case	BALB/c nude mice	HepG2, SK-HepG1, L02, HCCLM3, Huh7	miR-1299, CTNND1	–	↑CircMAST1, ↓miR-1299, ↑CTNND1: ↑HCC proliferation and migration	[79]
	hsa_circ_0056836		Nude mice	HUH7, SK-HEP-1, SNU449, HEPG2	miR-766-3p, FOSL2	–	↑hsa_circ_0056836, ↓miR-766-3p, ↑FOSL2: ↑ HCC proliferation and migration	[80]
Pancreatic cancer	Circ-PDE8A	PDAC = 93 case	Nude mice	BxPC-3, HEK-293, Capan-1, Aspc-1, Hs 766T, Hs 766T	-miR-338, MACC1 -MET signaling pathway	TNM stage, lymphatic invasion, poor survival rate	↑Circ-PDE8A, ↓miR-338, ↑MACC1, ↑MET/AKT, and ERK pathway: ↑ PDAC development and invasion	[81]
	Circ-ADAM9	PC tissue = 58 pairs	BALB/c nude mice	PANC1, HPDE, MiaPaca2, BxPC3, SW1990	miR-217, PRSS3	TNM stage, lymph node status	↑Circ-ADAM9, ↓miR-217, ↑PRSS3: ↑ PC malignancy and behavior	[41]
	ciRS-7	PDAC tissue = 41 pairs	–	PANC-1, BXPC-3, HPC-Y5	-miR-7 - EGFR/STAT3 signaling	Venous invasion, LNM	↑ciRS-7, ↓miR-7, ↑EGFR/STAT3 signaling: ↑ PC proliferation and metastasis	[82]
	Circ CDR1as	PC = 27 case	BALB/c nude mice	PC-3, HPDE6-C7, PANC1, BXPC-3, ASPC1, MIApaCa-2, CFPAC-1	miR-432-5p, E2F3	–	↑CircCDR1as, ↓miR-432-5p, ↑E2F3: ↑ PC progression	[83]
	hsa_circ_0006117	PC = 20 case	–	SW1990, PaCa-2, BxPC-3, PANC-1, MIA, AsPC-1	-miR-96-5p, -KRAS/MAPK Signaling	–	↑hsa_circ_0006117, ↓miR-96-5p, ↑KRAS/MAPK Signaling: ↑ PC progression	[43]
	CircBFAR	PDAC = 208 case	SCID mice	BxPC-3, hTERT-HPNE, MIA PaCa-2, PANC-1, CFPAC-1	-miR-34b-5p - MET/PI3K/Akt signaling	Gender, age, BMI, smoking, tumor size, differentiation, T stage, TNM stage, Ki67 expression, neoadjuvant chemotherapy, lymphatic metastasis, adjuvant chemotherapy	↑ CircBFAR, ↓ miR-34b-5p, ↑MET/PI3K/Akt signaling: ↑ PDAC progression	[42]
	hsa_circ_0014784	PC = 10 case	Nude mice	SW1990, HPDE6-C7, PANC-1, AsPC-1, BxPC3, Capan-2	miR-214-3p, YAP1	–	↑hsa_circ_0014784, ↓miR-214-3p, ↑YAP1: ↑ PC progression	[84]
	hsa_circ_0007367	Frozen PC tissue = 25 pairs	BALB/c nude mice	AsPC-1, HPDE, BxPC-3, SW1990, Capan-1, PANC-1	miR-6820-3p, YAP1	LNM, advanced histological grade	↑hsa_circ_0007367, ↓ miR-6820-3p, ↑ YAP1: ↑ PDAC progression	[85]

*PC* pancreatic cancer, *HCC* hepatocellular cancer, *GC* gastric cancer, *EC* esophageal cancer, *LPAR3* lysophosphatidic acid receptor 3, *Circ_LRP6* circular RNA-lipoprotein receptor 6, *CircPVT1* circular RNA plasmacytoma variant translocation 1, *Circ_RanGAP1* circular RNA_ran GTPase-activating protein 1, *CiRS-7* circular RNA sponge for miR-7, *CircDLG1* circular RNA discs large homolog 1, *CircSPARC* circular RNA secreted protein acidic and rich in cysteine, *CircSEMA5A* circular RNA semaphorin 5A, *Circ-METTL9* circular RNA methyltransferase like 9, *CircLDLR* circular RNA low-density lipoprotein receptor, *CircTMEM45A* circular RNA transmembrane protein 45A, *CircMAST1* circular RNA microtubule associated serine/threonine kinase 1, *Circ-PDE8A* circular RNA phosphodiesterase 8A, *Circ-ADAM9* circular RNA a disintegrin and metalloproteinase domain 9, *MET* mesenchymal epithelial transition, *RHOC* as homolog gene family member C, *RBBP7* retinoblastoma-binding protein 7, *VEGFA* vascular endothelial growth factor A, *DNMT3A* DNA methyltransferase 3 alpha, *JAK* Janus kinase, *STAT* signal transducer and activator of transcription, *NF-κB* nuclear factor kappa-light-chain-enhancer of activated B cells, *CDC25B* cell division cycle 25B, *CCNE1* cyclin E1, *CDK6* cyclin dependent kinase 6, *SOAT1* sterol o-acyltransferase 1, *NR2F6* nuclear receptor subfamily 2 group F member 6, *CDK4* cyclin dependent kinase 4, *Bcl2* B-cell lymphoma 2, *PKM2* pyruvate kinase muscle 2, *CTNND1* catenin delta 1, *MACC1* metastasis-associated in colon cancer 1, *PRSS3* protease, serine 3, *KRAS* kirsten rat sarcoma viral oncogene homolog, *YAP1* yes-associated protein 1.

**Fig. 2:**
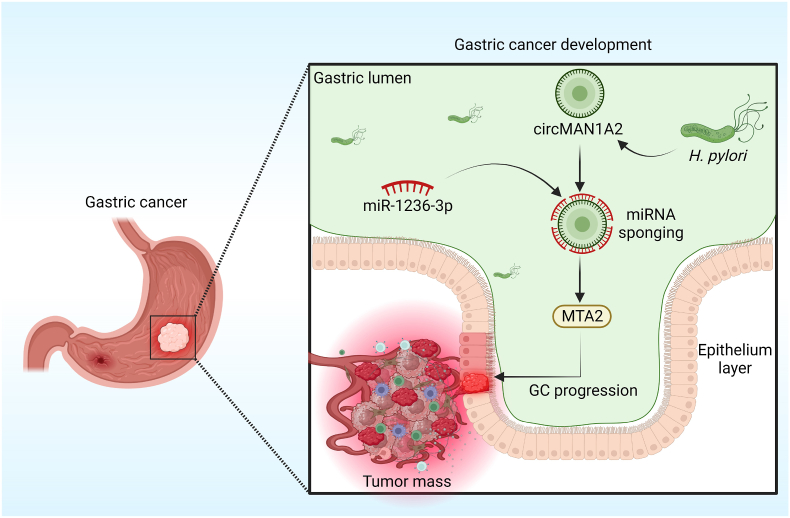
The diagram illustrates the interplay of circRNA and gut microbes in the progression of GC. CircMAN1A2 could induce GC progression, which was induced by H. pylori by miR-1236-3p sponging to regulate the expression of the MTA2 gene.

In contrast, in GI cancers, circRNAs have been found to inhibit tumor growth and offer great promise as therapeutic targets. These circRNAs act as tumor suppressors and have been found to be crucial in the development of chemoresistance in GI malignancies. They do this by influencing crucial signaling networks, affecting cellular processes, and regulating gene expression. For instance, circ-Foxo3 was shown to be reduced in ESCC cell lines and tissues, which suppresses ESCC growth via miR‐23a sponging and controls the PTEN gene expression [46]. Likewise, Fang et al. discovered that circFAT1(e2) controls tumor suppressor *RUNX1* expression in GC cells by acting as a sponge for miR-548 g, which in turn, by interacting with *YBX1* in the nucleus and targeting miR-548 g in the cytoplasm, suppresses the growth of GC [47]. Similarly, according to Chen et al., circRHOBTB3 expression is markedly downregulated in CRC tissues as well as cell lines, and it reduces the aggressiveness of the tumor through working with the HuR/PTBP1 axis [48]. Moreover, Zhong et al. demonstrated that circC3P1 works as a tumor suppressor by increasing *PCK1* expression via miR-4641 sponging in HCC [49]. Furthermore, Shi et al. confirmed that circa ANAPC7 is a new tumor suppressor that prevents muscle atrophy and tumor growth in PC by reducing cyclin D1 and TGF through the CREB-miR-373-PHLPP2 axis [50] (Fig. 2). Therefore, circRNAs could be identified and characterized to shed light on the molecular causes of GI cancer and possibly enhance therapeutic approaches. Table 2 lists the numerous circRNAs' tumor-suppressing functions in the development of GI tumors through controlling their target genes and signaling networks.

**Table 2:** Tumor suppressor roles of various circRNAs in GI tumorigenesis through regulation of target genes and signaling pathways (↑: upregulated, ↓: downregulated).

Cancer types	circRNAs	Clinical studies	Animal studies	Cell line studies	Target genes/signaling pathways	Clinicopathological characteristics	Description	Ref.
Esophageal cancer	Circ-Foxo3	ESCC tissues = 94 pairs	BALB/c nude mice	KYSE510, ECA109, TE‐1, TE‐13	miR-23a, PTEN	Age, TNM stage, gender, tumor size, histological grade	↓Circ-Foxo3, ↓ miR-23a, ↑PTEN: ↓ ESCC progression	[46]
	Circ-TNRC6B	-ESCC tissues = 53 - Healthy tissue = 48	–	TE-1, KYSE-170, KYSE-30, KYSE-150	miR-452-5p, DAG1	T stage	↓Circ-TNRC6B, ↓miR-452-5p, ↑DAG1: ↓ ESCC invasion and proliferation	[86]
	Circ_0007624	Tissue sample	–	–	-miR-224-5p, CPEB3 - EGFR/PI3K/AKT pathway	Poor prognosis	↓Circ_0007624, ↓miR-224-5p, ↑CPEB3, ↓ EGFR/PI3K/AKT pathway: ↓ ESCC development	[87]
Gastric cancer	CircFAT1(e2)	GC = 38 case	BALB/c mice	GSE-1, SGC-7901, AGS, BGC-823, MKN-28, MGC-803, MKN-45	miR-548 g/RUNX1	Overall survival	↓ CircFAT1(e2), ↓ miR-548 g, ↑ RUNX1: ↓ Tumor progression	[47]
	CircRHOBTB3	GC tissue = 75 pairs	BALB/C nude mice	AGS, MKN45, HGC27	-miR-654-3p - p21 signaling	Tumor stage	↓CircRHOBTB3, ↓miRNA-654-3p, ↑p21 signaling: ↓ GC growth	[88]
	CircRTN4	Tissue sample	–	–	miR-424-5p, LATS2	Low survival rate	↑CircRTN4, ↓miR-424-5p, ↓LATS2: ↓ GC development	[89]
	circ-HN1	GC tissue = 30 pairs	–	GES-1, MKN-28, HGC-27, SGC-7901, AGS	miR-485-5p, GSK3A	–	↓Circ-HN1, ↓miR-485-5p, ↑GSK3A: ↓ GC progression	[90]
	CircEIF4G3	-GC tissue = 103 pairs - Serum = 120 from GC individuals - Serum = 50 from gastritis individuals - Serum = 120 normal controls	BALB/c nude mice	AGS, GSE-1, HGC-27, MKN-45, HEK-293 T, SGC-7901	miR-4449, SIK1, β-catenin pathway	TNM stage, venous invasion	↑CircEIF4G3, ↓miR-4449, ↑SIK1, ↓β-catenin pathway: ↓ GC development and metastasis	[91]
	hsa_circ_0026344	–	–	–	miR-590-5p, PDCD4	Tumor size, TNM stage, LNM	↓hsa_circ_0026344, ↓miR-590-5p, ↑PDCD4: ↓GC progression	[92]
	CircPFKP	GC tissue = 25 pairs	Nude mice	HGC-27, GES-1, MKN45, NCI–N87, SGC-7901, AGS	miR-644, ADAMTSL5	–	↑CircPFKP, ↓ miR-644, ↑ADAMTSL5: ↓ GC metastasis and proliferation	[93]
Colorectal cancer	CircRHOBTB3	CRC = 83 pairs	BALB/c nude mice	RKO, NCM460, HCT116, FHC, SW480, HT29, SW620, DLD-1, HCE8693, Colo320	HuR/PTBP1	Advanced clinical stages and greater risk of metastases	↓CircRHOBTB3, ↓ PTBP1: ↓ metastasis	[48]
	Circ0104103	Tissue samples	BALB/c nude mice	NCM460, CACO2, HCT116, HT29, DLD1, HCT8, SW620, SW480	LACTB, miR‐373‐5p	TNM stage, tumor invasion depth	↓Circ0104103, ↓miR‐373‐5p, ↑LACTB: ↓ CRC progression	[94]
	CircCUL2	–	Nude mice	HT290, FHC, HCT116, SW620, SW480	miR-208a-3p, PPP6C	TNM stage and distant metastasis	↓CircCUL2, ↓miR-208a-3p, ↑PPP6C: ↓ CRC proliferation	[95]
	CircITGA7	–	Mice	SW480, FHC, RKO, DLD1, Caco-2, HCT116, LoVo, SW620	-miR-370-3p, ITGA7, RREB1 -Ras signaling	TNM stage, tumor size, distant metastasis, lymph metastasis	↓CircITGA7, miR-370-3p, ↑ITGA7, ↓ RREB1, ↓ Ras signaling: ↓CRC development and metastasis	[96]
Hepatocellular carcinoma	CircDLC1	HCC = 110 case	BALB/c nude mice	Huh-7, SNU449, Hep3B, SK-Hep1, HepG2	MMP1	AFP level, BCLC stage, TNM stage, macrovascular invasion, microvascular invasion, OS, RFS	↓CircDLC1, ↓MMP1: ↓ HCC progression	[97]
	CircMTO1	HCC tissue = 289 pairs	Nude mice	HepG2, SK-Hep1, QGY-7701, SMMC-7721,	miR-9, p21	–	↓CircMTO1, ↓miR-9, ↑p21: ↓ HCC progression	[98]
	cSMARCA5	HCC tissue = 208 pairs	BALB/c nude mice	HCCLM3, Huh7, Hep3B, SMMC-7721, MHCC97H	miR-17-3p, miR-181b-5p, TIMP3	Poorer tumor differentiation, microvascular invasion, advanced tumor stage, tumor size, OS	↓cSMARCA5, ↓miR-17-3p and miR-181b-5p, ↑TIMP3: ↓ HCC development and metastasis	[99]
	CircC3P1	HCC tissue = 47 pairs	BALB/c nude mice	BEL7402, HL-7702, Hep3B, MHCC97-L, HuH7	miR-4641, PCK1	Age, gender, AFP, size, TNM, vascular invasion	↓CircC3P1, ↓miR-4641, ↑PCK1: ↓ HCC development and metastasis	[49]
	CircTRIM33–12	HCC = 150 case	Nude mice	SMMC-7721, HCCLM3, Huh 7, HepG2, MHCC97L	miR-191, TET1	Poor prognosis	↓CircTRIM33–12, ↓miR-191, ↑TET1: ↓ HCC progression and metastasis	[100]
Pancreatic cancer	CircANAPC7	–	Athymic nude mice	AsPC-1, HPDE, MIA PaCa-2, CFPAC-1, Panc-1, BxPC-3	CREB, miR-373, PHLPP2	–	↑CircANAPC7, ↓miR-373, ↓CREB, ↓PHLPP2: ↓ PC tumor growth and muscle wasting	[50]
	Circ_0047744	Tissue samples	–	HPDE6-c7, PANC-1	miR-21, SOCS5	LNM, positively correlated with OS	↓circ_0047744, ↓miR-21, ↑SOCS5: ↓PDAC metastasis	[101]
	Circ-STK39	Tissue sample	–	–	miR-140-3p, TRAM2	–	↓Circ-STK39, ↓ ↓miR-140-3p, ↓TRAM2: ↓ PC progression	[102]
	hsa_circRNA_001587	PC tissue = 67 pairs	BALB/C nude mice	HPDE, PC-3, AsPC-1, COLO357, PANC-1	miR-223, SLC4A4, MMP-2, MMP-9	Tumor differentiation, LNM	↓hsa_circRNA_001587, ↓miR-223, ↑SLC4A4: ↓ PC angiogenesis, migration and invasion	[103]

*Circ-Foxo3* circular RNA forkhead box O3, *Circ-TNRC6B* circular RNA trinucleotide repeat containing adaptor 6B, *CircRTN4* circular RNA reticulon 4, *CircEIF4G3* circular RNA enhancer of eIF4G3, *CircITGA7* circular RNA integrin subunit alpha 7, *CircMTO1* circular RNA mitochondrial translation optimization 1 homolog, *Circ-STK39* circular RNA serine/threonine kinase 39, *PTEN* phosphatase and tensin homolog, *PI3K* phosphoinositide 3-kinase, *RUNX1* runt-related transcription factor 1, *SIK1* salt inducible kinase 1, *PTBP1* polypyrimidine tract-binding protein 1, *LACTB* lactamase beta, *PPP6C* protein phosphatase 6 catalytic subunit, *ITGA7* integrin subunit alpha 7, *MMP1* matrix metallopeptidase 1, *TIMP3* tissue inhibitor of metalloproteinases-3, *PCK1* phosphoenolpyruvate carboxykinase 1, *TET1* tet methylcytosine dioxygenase 1, *TRAM2* translocating chain-associated membrane protein 2.

## 3 Role of gut microbiome in GI cancers

The gut microbiota may affect GI cancer progression through several mechanisms, including alteration of immune function, modification of gut barrier activity, and production of carcinogenic metabolites [19]. Several GI malignancies, including EC, CRC, GC, HCC, and PC, have been linked to an elevated risk because of modifications in the structure and activity of the gut microbiota [104]. The efficiency of anticancer treatments like chemotherapy and immunotherapy may also be influenced by the gut microbiota [105].

Moreover, FMT stands for a prospective cancer treatment plan through improving bile acid metabolism, reestablishing the gut microbiota, and adjusting the effectiveness of immunotherapy [106]. Gut dysbiosis can be brought on by a variety of causes, including host genetics, nutrition, antibiotics, and stress [107]. Through the activation of tumorigenic pathways, generating inflammation, and harming host DNA, microbial dysbiosis and specific bacteria in the gut might influence the growth and progression of cancer [108]. Certain bacterial products, such as the CagA protein of *Helicobacter pylori* [109], the FadA toxin of *Fusobacterium nucleatum* [110], the AvrA protein of *S.enterica Typhi* [111], and BFT from *Enterotoxigenic Bacteroides fragilis*, may encourage E-cadherin and β-catenin separation, which can result in β-catenin activation and aid in tumor development [112]. Microbial dysbiosis also results in a reduction in the beneficial component of bacterial metabolites such as short-chain fatty acids (SCFAs) [113]. The microbe-associated molecular patterns (MAMPs), which activate TLRs in macrophages and dendritic cells, play a role in intestinal dysbiosis's potential for bacterial translocation and exert a pro-inflammatory consequence [114]. TLR signaling encourages the expression of pro-inflammatory molecules like IL-23, TNF, and IL-1, which promote the growth of cancer [115]. Moreover, numerous microbial metabolites can directly or indirectly harm host DNA, which promotes tumorigenesis. Special microbial toxins, such as CDT and colibactin, may cause DNA damage directly [116]. In addition, DNA is also indirectly harmed by gut bacteria via polyamines, DCA, RNS, ROS, and H2S [117] (Fig. 1).

*BFT* bacteroides fragilis toxin; *FadA* fusobacterium adhesin A; *CAM* cell adhesion molecule; *IFN* interferon; *LPS* lipopolysaccharide; *NF-κB* nuclear factor-κB; *MAMP* microbe-associated molecular pattern; *STAT3* signal transducer and activator of transcription 3; *PRR* pattern recognition receptor; *TLR4* Toll-like receptor 4.

## 4 The interplay between circular RNAs and microbiota in gastrointestinal cancer

The circRNAs and the microbiota interaction in GI cancer is a growing area of research that might provide information on the intricate pathways that underlie the development of cancer. *Helicobacter pylori* is recognized as a major contributor to the development of stomach cancer [118]. In GC, Guo et al. found that independent of CagA, *Helicobacter pylori* could cause the overexpression of circMAN1A2 in AGS and BGC823 cells to induce GC tumorigenesis [119]. They showed that *Helicobacter pylori* might cause CagA-independent overexpression of circ_MAN1A2 in GC cells. Furthermore, in vivo, the growth of xenograft tumors was decreased by circ_MAN1A2 knockdown, and the advancement of GC was linked to circ_MAN1A2 overexpression (Fig. 2). Thus, circ_MAN1A2 may be a new GC therapeutic target and diagnostic marker.

Similarly, Diling et al., found that the gut microbiota and brain circRNA sequence profiles of AD-like animals were altered due to circNF1-419's ability to improve autophagy and treat senile dementia. According to their findings, over-expression of circNF1-419 in the brain altered the composition of the gut microbiota, including *Candidatus arthromitus*, *Lachnospiraceae FCS020* group, *Lachnospiraceae UCG-006* group, and [*Eubacterium*] *Xylanophilum* group, as well as intestinal homeostasis and physiology, and even the progression of the gut microbiota in newborn mice [120].

In contrast, tumor metastasis is controlled by gut microbiota through the circRNA and miRNA pathway. For example, it was proved that the specified pathogen-free (SPF) mice treated with ABX showed increased lung metastases. Lung metastases was dramatically reduced after fecal microbiota transplantation (FMT) from SPF mice or Bifidobacterium into germ-free mice. In order to control the concentrations of oncogenic miRNAs, the gut microbiota influences the expression of circRNA such as mmu_circ_0000730 [121]. In particular, these modifications in the gut microbiota suppress the expression of IL-11. Mutual suppression between mmu circ_0000730 and mmu-miR-466i-3p was identified using bioinformatics tools and luciferase reporter studies. Internal homeostasis and appropriate protection against microbial infection and malignancy are two examples of how circRNAs contribute to human immunity (Table 3).

**Table 3:** The circRNAs and the microbiota interaction in GI malignancies.

CircRNA	Associated microbes	Targes	Cell line	Animal	Clinical sample	Findings	Ref.
CircMAN1A2	*Helicobacter pylori*	miR-1236-3p, MTA2	GES-1, AGS, BGC823	BALB/c nude mice	Hp− human gastritis = 52, Hp + human gastritis = 47	By sponging miR-1236-3p to control the expression of MTA2, circMAN1A2 could accelerate the development of GC brought on by H. pylori.	[119]
CircNF1-419	*Candidatus Arthromitus, Lachnospiraceae UCG-006, Lachnospiraceae FCS020 group,* and [Eubacterium] *xylanophilum group*	**-**	**-**	SAMP8 mice	**-**	In AD-like mice, CircNF1-419 enhances gut microbiota structure and function.	[120]
ciRS-7	*Cryptosporidium parvum*	NF–B signaling pathway	HCT-8	**-**	**-**	CiRS-7 might facilitate the spread of C. parvum by controlling the miR-1270/relA axis and the NF–B pathway.	[122]
mmu_circ_0000730	**-**	mmu-miR-466i-3p, mmu-miR-466 f-3p, SOX9, IL-11	LLC, B16–F10	C57BL/6 mice	**-**	Through the IL-11/circRNA/miRNA/SOX9 axis, gut microbiota controls the development and metastasis of cancer.	[121]
circHIPK2	*Firmicutes, Proteobacteria, Bacteroidetes*	**-**	**-**	NLRP3 KO mice	**-**	NLRP3-deficient mice's gut microbiome reduces depressive-like behaviors through controlling astrocyte dysfunction via circ HIPK2	[123]

However, understanding the complex relationships between these two variables may lead to the development of new therapeutic approaches and biomarkers, ultimately improving the detection, diagnosis, and treatment of GI malignancies.

## 5 Gastrointestinal cancer and therapeutic targets

GI malignancies are a group of tumors that generally affect numerous digestive system organs. Numerous therapeutic targets for GI tumors have been discovered over time, and targeted medicines are now a crucial component of cancer treatment. Here are several treatment approaches for GI malignancies that are frequently researched.

## 6 Current treatment strategies for gastrointestinal cancers

According to the specific kind and stage of the tumor, many therapeutic approaches are used for GI malignancies [124]. Surgery, chemotherapy, radiation therapy, targeted treatments, and immunotherapy are frequently used as treatment options [125,126].

For localized GI malignancies, surgery is frequently the first line of treatment [127]. To stop the spread of cancer cells, the tumor and adjacent lymph nodes must be removed [128]. If the cancer is advanced or has spread to distant organs, surgery may not always be an option [129]. Similarly, chemotherapy is a systemic therapy that employs medications to either kill or limit the growth of cancer cells. When surgery is not an option, chemotherapy is frequently employed before or after surgery to reduce tumor size [130]. GI malignancies are routinely treated with combination chemotherapy regimens [131]. Likewise, to eradicate cancerous cells and reduce tumors, radiation treatment employs high-energy photons. It can be used as the main course of treatment for localized GI malignancies, in conjunction with surgery or chemotherapy, or to treat advanced cases of the disease's symptoms [132]. Moreover, drugs used in targeted therapy precisely target certain molecules or pathways thought to be essential in the progression and spread of tumors [133]. For instance, HER2-targeted treatments in PC [134], VEGF inhibitors [135], and EGFR inhibitors [136] are utilized to treat particular GI malignancies. In addition, through immunotherapy, cancer cells are recognized and attacked by the patient's immune system. Some GI malignancies have responded well to the use of immune checkpoint inhibitors, particularly in patients with advanced or metastatic illness [137]. Examples of these agents are *PD-1* and *PD-L1* inhibitors [138]. Furthermore, neoadjuvant therapy is another therapeutic approach used to reduce tumors before surgery and enhance its results [139]. Following surgery, adjuvant therapy is administered to lower the possibility of a cancer recurrence [140].

Despite that, healthcare is a crucial part of treatment for those with advanced or metastatic GI malignancies [141]. Through the control of symptoms, pain, and other side effects of malignant therapy, healthcare aims to increase the quality of life [142].

Thus, the specifics of the tumor, the cancer's stage, and the patient's general health all play a role in determining the course of treatment for GI malignancies. For each patient, multidisciplinary teams of oncologists, surgeons, radiation oncologists, and other professionals collaborate need to develop the most efficient and individualized course of therapy.

## 7 Strategies to target circRNAs

In recent years, significant advancements have been achieved in the research of circRNA. A growing body of research has confirmed that circRNAs can be therapeutically targeted in both vivo and in vitro using a range of methods, including exosome-mediated transport, RNA interference (RNAi), ASOs, and circRNA modification. Even though research in this area is still ongoing, here are some potential strategies that researchers are taking into consideration.

A strategy based on siRNAs makes use of the endogenous RNAi process, in which double-stranded RNA (dsRNA) molecules cause post-transcriptional silencing [143]. Short interfering RNA (siRNA) or short hairpin RNA are frequently used in RNAi to knock down circRNAs. By complementary pairing, siRNAs, which are 21–23 nt in dsRNA length, target circRNAs and add them to the RISC, where they will be cleaved [30]. ShRNAs are converted into siRNAs after being processed and are distinguished by their loops and base-paired stems [144]. The back-splice junction particular to circRNAs is typically targeted to reduce circRNAs without impacting their corresponding linear mRNA. Further, through complementary pairing, antisense oligonucleotides (AON) can also target circRNAs [145]. Despite their effectiveness at blocking protein interaction sites on circRNAs, their length prevents them from being used to specifically target the back-splice junction and knockdown of circRNAs. In addition, the most practical approach to knocking down circRNAs in vivo currently involves the use of siRNA and shRNA delivered in lipid-based polymers. Nevertheless, RNAi molecules have some drawbacks that need to be addressed. These include their quick destruction by nucleases [146], inefficient cellular delivery [147], lack of cell selectivity [148], immunogenicity [149], and off-target consequences [150].

Moreover, direct biogenesis and purification allow circRNAs to be overexpressed [151]. Many different methods can be used to circularize RNA [152]. Splint ligation can be used to cycle single-stranded linear RNA that has been chemically or in vitro transcribed [153]. This approach produces exceedingly pure circRNA molecules that can be given directly to target cells [154]. Also, a successful in vitro miRNA sponge has been produced using this technique [155].

By employing nanoparticles as carriers, circRNA molecules can be efficiently delivered to specific cells or tissues while simultaneously being protected, resolving several concerns with conventional therapies [156]. After being released into the tumor target cells, circRNA-based medicines can start to work in several different ways [157]. They can act as powerful regulators, influencing signaling pathways, changing gene expression, or absorbing microRNAs, all of which have the potential to have therapeutic effects [158]. For instance, Wang et al. proved that using nanoparticles loaded with circ 0086375 to target the miR-646/SLC4A4 axis can prevent PC from getting worse. In which circ 0086375 was identified as miR-646's verified target, it worked as a miR-646 sponge to elevate the expression of *SLC4A4* [159]. Similarly, You et al. showed that has-circ-0058051 may function as an oncogene that induces HCC cell proliferation and migration. They developed a safe and effective magnetic nanoparticle-mediated delivery strategy for transporting circ-0058051-siRNA under an external MF to silence circ-0058051 in HCC. Their outcomes demonstrated that si-circ-0058051 is protected effectively by PEG-PCL-PEI-C14-SPIONs (PPPCSs) against degradation by serum and tissue enzymes. As a result, circ_0058051 was silenced in vitro and in vivo by the PPPCSs/si-circ_0058051 complex, which greatly reduced carcinogenesis and HCC development [160].

**Fig. 3:**
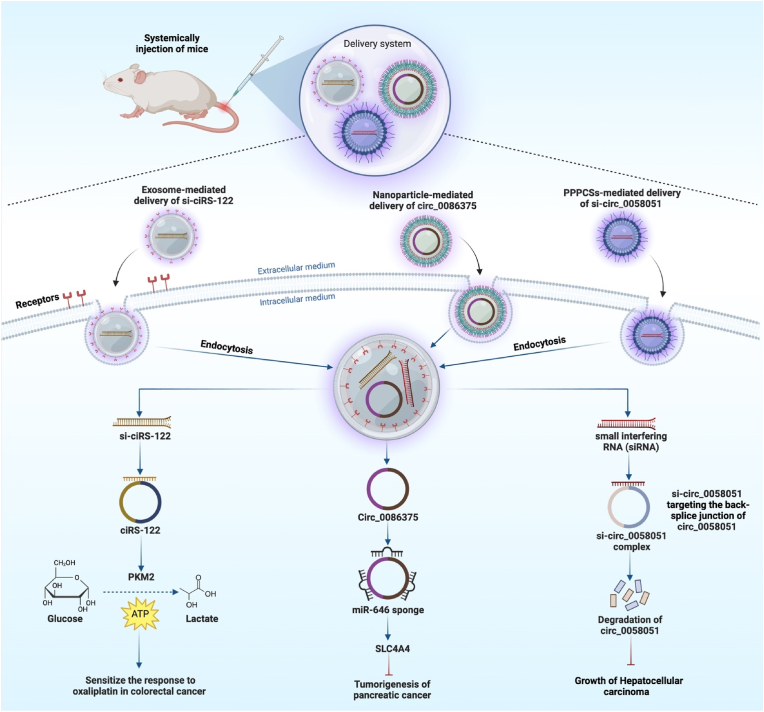
The illustration shows common ways to target circRNAs in vivo as a therapeutic approach to knocking down circRNAs, such as exosome-induced delivery of siRNA and nanoparticle-induced delivery of siRNA.

In addition to nanoparticles, exosomes, which are small extracellular vesicles made by cells, can be used to naturally transport circRNAs [161]. When exosomes are taken up by the recipient cells, the circRNAs are sent straight to the cytoplasm [162]. The circRNAs can either interact with RBPs or act as sponges for miRNAs to influence gene expression and signaling pathways linked to drug resistance or other pathogenic events [163]. For instance, Wang et al. found that CRC cell exosomes contained an enriched form of ciRS-122, which contributed to therapeutic resistance by miR-122 sponging and overexpressing PKM2. The increased PKM2 led to higher glycolysis and ATP production, allowing cells to expel drugs effectively. The chemoresistant CRC cells transferred si-ciRS-122 via exosomes to nonresistant cells to sensitize the response to oxaliplatin in CRC by preventing glycolysis [164] (Fig. 3).

Further, CRISPR/Cas gene-editing system is a cutting-edge tool for precisely targeting and silencing circular RNA molecules through a process called CRISPR/Cas-mediated circRNA knockdown [165]. The ability to knock down circRNAs with CRISPR/Cas is a powerful method for studying the roles of these non-coding RNAs in disease [166]. To achieve circRNA knockdown, the CRISPR/Cas system should be designed to target the back-splice junction region of the circular RNA molecule [167]. CRISPR/Cas is a particularly useful tool for circRNA knockdown because of its high specificity and flexibility, which allow precise targeting of individual circRNAs in a cell- or tissue-specific way [168].

## 8 Targeting the gut microbiome in GI cancer therapy

A developing area of inquiry that holds promise as a potential therapeutic method is targeting the gut microbiota in the treatment of GI [169]. The microbiome of the gut is an important component in the processes that keep gut health, digestion, and immunity in good standing [170,171]. Growing evidence suggests that the gut microbiota influences cancer progression and the efficacy of cancer therapy [172]. Various studies have shown that the presence or absence of certain gut bacteria can promote or inhibit tumor growth, as well as influence how well chemotherapy and immunotherapy work [173].

**Fig. 4:**
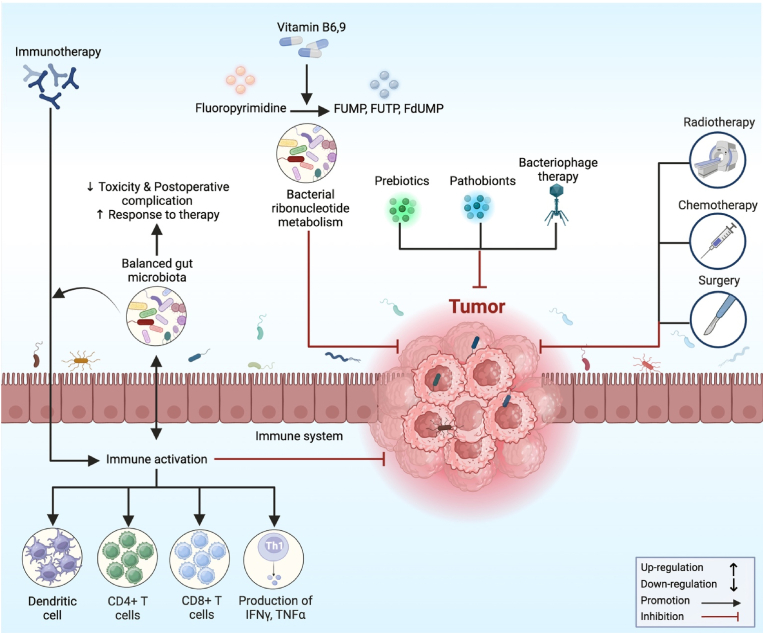
The schematic diagram illustrates the multifaceted mechanisms of microbiota modulation in response to therapy aimed at suppressing tumor growth. These complex mechanisms entail the gut microbiota's dynamic interaction with the host's immune system, therapeutic drugs, and the tumor environment.

Scientists are looking at the potential benefits of gut microbiota in the diagnosis and intervention of gastrointestinal cancer. For instance, oncologists may be able to personalize cancer treatment strategies by having a better understanding of a patient's gut microbiota [174]. By analyzing a patient's gut microbiota, medical professionals may be able to predict the patient's response to certain drugs [175]. Moreover, in CRC, numerous strategies have been demonstrated to target and alter the composition of the gut microbiota, considering both microbial physiology and/or their metabolites that directly or indirectly lead to CRC [176]. These strategies include dietary interventions, antibiotic treatments, probiotics, prebiotics, and postbiotics, as well as FMT [177] (Fig. 4). For instance, through several processes, including microbial-derived elements such as metabolites or genotoxins, the gut microbiota can affect the growth of CRC [178]. Antibiotic use is effective at eliminating pathobionts, but because it also kills beneficial bacteria for health, it hurts gut homeostasis and should therefore be used less frequently in CRC care [179].

Probiotic development is encouraged by prebiotic activity. Probiotics have a variety of anti-cancerogenic effects, including the prevention of pathogenic bacteria from colonizing the body [180], improving barrier function by increasing the production of mucin and tight junction proteins [181], encouraging homeostatic immune responses by encouraging the growth of anti-inflammatory responses by Treg cells [182], modulating pro-inflammatory cytokine release [183], and encouraging cancer cell apoptosis. Additionally, postbiotics cause selective cytotoxicity against tumor cells as well as the regulation of tumor cell proliferation [184].

Furthermore, FMT has the potential to be employed in the treatment of GI cancers by preventing the colonization of pathogenic bacteria [185]. This method has demonstrated promise in treating specific digestive disorders, such as recurrent *Clostridium difficile* infections [186,187]. The growing interest in the potential of FMT for the treatment of cancer is sparked by the observation that cancer patients often have intestinal dysbiosis [188]. Thus, to develop novel and individualized therapeutic strategies in the fight against GI malignancies, additional study is required to better understand the complex relationships between the gut microbiome and cancer.

## 9 Microbiota-targeted circRNA therapies

Microbiota-targeted circRNA therapies are an innovative approach to personalized medicine that hopes to focus on the connections between the gut microbiota and human health. Recent studies have revealed how circRNAs affect host-microbiota crosstalk, which affects physiological processes and the onset of illness [29,189]. Through circRNA-based interventions, these treatments aim to modify the structure and function of the gut microbiota. Additionally, bacteria have emerged as a possible cancer treatment tool due to their natural affinity for hypoxic tumor environments and their potential for genetic engineering as a vector for gene and medication therapy [190].

Microbiota-targeted circRNA treatments work by identifying and modifying certain circRNAs that serve as brokers of host-microbiota interactions [191]. These circRNAs may serve as signaling pathway regulators, binding partners, or molecular scaffolds in the homeostasis of the microbiota [192,193]. Understanding the intricate interactions between circRNAs and the gut microbiota will allow researchers to create artificial circRNAs or alter endogenous ones to precisely control the structure and activity of the microbiota. Innovative delivery methods, such as nanoparticles, synthetic bacteria, or even genetically altered probiotics, are used to deliver circRNA therapeutics to the gut [194,195]. In the gut environment, where they may exert their regulatory effects on the microbiota, these vehicles ensure the exact release of circRNAs. By using this method, circRNA treatments may be able to reduce inflammation, dysbiosis, and other conditions that are impacted by the microbiome.

**Fig. 5:**
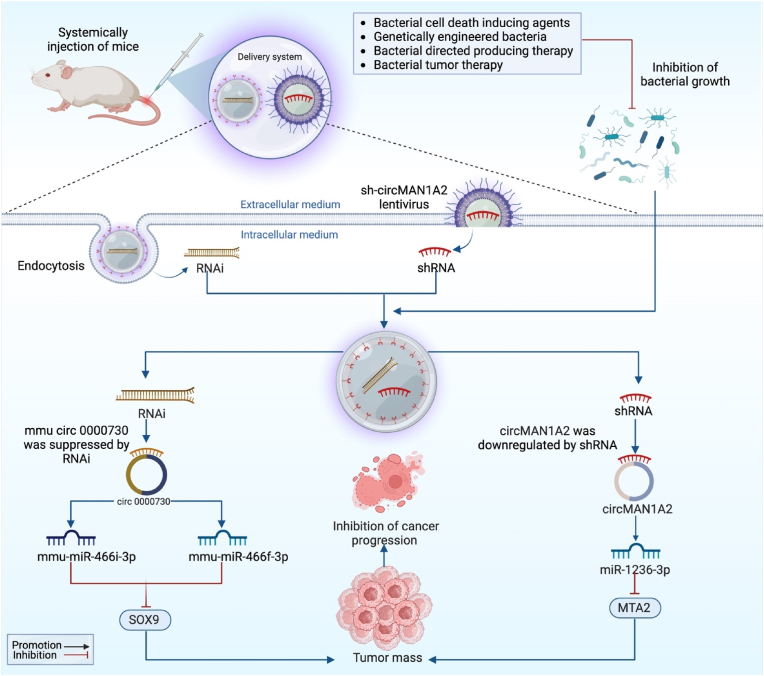
The schematic illustration represents the microbiota-targeted circRNA therapies. Through the systematic administration of RNAi and shRNA, and the inhibition of microbial growth, circ000730 and circMAN1A2, as well as their corresponding target genes SOX9 and MTA2, are downregulated, which inhibits the advancement of cancer.

For instance, expression of mmu-miR-466i-3p and mmu-miR-466f-3p was dramatically elevated when mmu circ 0000730 was suppressed by RNAi. Through modulation of the gut microbiota SOX9 or mmu circ 0000730 dramatically downregulated and decreased cancer cell invasion. Thus, mmu_circ_0000730 targets mmu-miR-466i-3p and inhibits cancer progression by decreasing SOX9 expression and blocking the STAT3 signaling pathway [121]. Similarly, in gastric cancer, Guo et al. revealed that downregulation of circMAN1A2 prevents the growth of gastric tumor cells. Therefore, to determine how circMAN1A2 affected the development of gastric cancer in vivo, they used a xenograft mouse model. By using sh-circMAN1A2 lentivirus and inhibiting *H. pylori* growth, they reduced circMAN1A2 expression in BGC823 cells. Downregulating circMAN1A2 expression significantly slowed tumor development, weight, and volume as compared to the control group by sponging miR-1236-3p to regulate MTA2 expression. These findings imply that downregulating circMAN1A2 can prevent gastric cancer cells from proliferating in vivo [119] (Fig. 5).

However, exploring combination therapies that simultaneously target the gut microbiome and circRNAs may have synergistic effects and open up new therapeutic possibilities for the treatment of GI cancer.

## 10 CircRNAs and gut microbiota as indicators for GI cancer prognosis and diagnosis

The gut microbiota and circRNAs play significantly important roles in the landscape of GI malignancies as indications for prognosis and diagnosis [29]. CircRNAs, which were once thought to be splicing artifacts, have emerged as powerful molecular markers as a result of their unique characteristics, such as stability and tissue-specific expression [196]. These characteristics make them promising candidates for prognostic assessment, as changed expression profiles of particular circRNAs have been linked with various disease stages, recurrence, and overall patient survival [197].

Interestingly, circRNAs have the potential to be used as a diagnostic tool, as their abnormal patterns of expression can be used to differentiate between malignant tissues and their healthy counterparts or normal tissues [198]. For instance, Wang et al. demonstrated that circSLIT2 RNA accumulation was higher in GC tissues compared to non-tumor tissues and was associated with distant tumor metastases, and plasma circSLIT2 was only found in GC patients. Plasma circSLIT2 positively correlated with circSLIT2 in GC tissues, distinguishing GC patients from other disease groups and HC patients [199]. Similarly, Wang with his colleagues confirmed that tissue and plasma expression levels of circ_0071662 were found to be decreased in HCC patients compared to HCs and other patient groups. This decreased expression was associated with poor survival, tumor metastasis, and tumor size, and was only observed in the radioresistance group after radiotherapy [200]. Likewise, in CRC, Li et al. demonstrated circGAPVD1's diagnostic efficacy in plasma exosomes. They expected that the highly expressed circGAPVD1 will serve as a novel CRC diagnostic marker [201].

Moreover, the GI tract's complex ecosystem, the gut microbiota, has become recognized as a major factor in processes that are connected to cancer [202]. The complex interactions between the host's health and the gut microbiota have made it a valuable source of predictive data [203]. Patients with GI cancer have had a range of outcomes, and variations in microbial compositions have been linked to these outcomes, offering information on the course of the disease and the survival of the patient [204]. These microbial signatures may be used as prognostic markers, assisting in the customization of treatment plans based on unique patient profiles. Additionally, the gut microbiota's impact on immune responses and metabolism affects fecal metabolites and immunological markers, paving the way for non-invasive diagnostic techniques that could completely transform the early identification of cancer [205]. According to Guo et al. study, *Fusobacterium nucleatum* may contribute to microbiota dysbiosis by secreting compounds that are hostile to probiotics. Furthermore, they was discovered that the ratio of *Fusobacterium nucleatum* to the crucial probiotics *Faecalibacterium prausnitzii* and *Bifidobacterium* is a useful biomarker for early CRC screening [206]. Similarly, in GC, salivary *Fusobacterium nucleatum* abundance shows promise as a biomarker for the diagnosis of GC, and *Fusobacterium nucleatum* infection may accelerate the EMT process and cause GC metastases [207]. CircRNAs and gut microbiota are expanding as promising biomarker for GI cancer prognosis and diagnosis. However, despite their enormous potential, many strategies are still being researched and might not have been widely used in clinical practice yet.

## 11 Conclusion

GI malignancies' relationship with circRNAs and the gut microbiota has become a novel field of study with important implications for comprehending carcinogenesis and identifying possible treatment targets. CircRNAs, a type of ncRNA, have been discovered to have essential roles in controlling many cellular processes associated with the initiation and development of GI malignancies. Additionally, it is now widely acknowledged that the trillions of microbes that survive in the gastrointestinal system and make up the gut microbiome have an essential role in controlling host physiology, immunological responses, and even cancer development.

The discovery of prospective treatment targets has been made possible by a better understanding of the involvement of circRNAs and the gut microbiome as well as their interactions in GI malignancies. It may be possible to develop new methods for cancer treatment and detection by focusing on particular circRNAs linked to carcinogenesis. Despite that, using probiotics, prebiotics, or FMT to modify the gut microbiome may provide creative strategies to affect circRNA-mediated cancer-related pathways.

A promising route for expanding our knowledge of cancer biology and creating new therapeutic approaches is the investigation of circRNAs and the gut microbiota in GI malignancies. Future advancements in cancer treatment and patient outcomes could result from continued study in this field. However, it's important to recognize that this area of study is still in its early stage and further study is needed to fully understand the complex mechanisms behind the interaction between circRNAs and the gut microbiota in GI malignancies.

## Ethics approval and consent to participant

Not applicable.

## Consent of publication

Not applicable.

## Availability of data and materials

Not applicable.

## Competing interest

The authors declare they have no conflict of interest.

## Funding

Not applicable.

## CRediT authorship contribution statement

**Sara Tharwat Abdullah:** Data curation, Investigation, Visualization. **Snur Rasool Abdullah:** Data curation, Methodology, Supervision. **Bashdar Mahmud Hussen:** Data curation, Validation. **Yousif Mohammed Younis:** Investigation, Methodology, Writing – original draft. **Mohammed Fatih Rasul:** Validation, Writing – original draft, Writing – review & editing. **Mohammad Taheri:** Investigation, Resources, Supervision.

## Abbreviation

Bcl2: B-cell lymphoma 2
CD274: Cluster of Differentiation 274
CDC25B: Cell division cycle 25B
CDK6: Cyclin-dependent kinase 6
c-Myc: Cellular-myelocytomatosis oncogene
CREB: cAMP-response element binding protein
CTNND1: Catenin delta 1
DNMT3A: DNA methyltransferase 3 alpha
FBXO22: F-box protein 22
FGFR1: Fibroblast growth factor receptor 1
FNDC3B: Fibronectin type III domain containing 3B
FOXO3: Forkhead box O3
HK2: Hexokinase 2
IGF2: Insulin-like growth factor 2
IL-10: Interleukin 10
ITGA7: Integrin subunit alpha 7
JAK: Janus kinase
KRAS: Kirsten rat sarcoma viral oncogene homolog
LACTB: Lactamase beta
MMP-2: Matrix metalloproteinase-2
MMP-9: Matrix metalloproteinase −9
mTOR: echanistic target of rapamycin
NF-κB: Nuclear factor kappa-light-chain-enhancer of activated B cells
NR2F6: Nuclear receptor subfamily 2 group F member 6
PCK1: Phosphoenolpyruvate carboxykinase 1
PIK3R1: Phosphoinositide-3-kinase regulatory subunit 1
PKM2: Pyruvate kinase muscle 2
PPP6C: Protein phosphatase 6 catalytic subunit
PTBP1: Polypyrimidine tract-binding protein 1
PTEN: Phosphatase and tensin homolog
RBBP7: Retinoblastoma-binding protein 7
RREB1: Ras-responsive element-binding protein 1
SLC4A4: Solute carrier family 4-member 4
SOCS5: Suppressor of cytokine signaling 5
SPARCL1: SPARC-like protein 1
STAT3: Signal transducer and activator of transcription 3
TCF7: Transcription factor 7
TRAM2: Translocating chain-associated membrane protein 2
VEGFA: Vascular endothelial growth factor-A
YAP1: Yes-associated protein 1
